# Cracking arbitrariness: A data-driven study of auditory iconicity in spoken English

**DOI:** 10.3758/s13423-024-02630-0

**Published:** 2025-01-08

**Authors:** Andrea Gregor de Varda, Marco Marelli

**Affiliations:** Department of Psychology, University of Milano – Bicocca, Piazza dell’Ateneo Nuovo 1, Milan, MI 20126 Italy

**Keywords:** Iconicity, Onomatopoeia, Phonosymbolism, Iconicity ratings, Deep learning, Computational modeling

## Abstract

Auditory iconic words display a phonological profile that imitates their referents’ sounds. Traditionally, those words are thought to constitute a minor portion of the auditory lexicon. In this article, we challenge this assumption by assessing the pervasiveness of onomatopoeia in the English auditory vocabulary through a novel data-driven procedure. We embed spoken words and natural sounds into a shared auditory space through (a) a short-time Fourier transform, (b) a convolutional neural network trained to classify sounds, and (c) a network trained on speech recognition. Then, we employ the obtained vector representations to measure their objective auditory resemblance. These similarity indexes show that imitation is not limited to some circumscribed semantic categories, but instead can be considered as a widespread mechanism underlying the structure of the English auditory vocabulary. We finally empirically validate our similarity indexes as measures of iconicity against human judgments.

## Introduction

Why are objects called the way they are? The traditional perspective on the structure of the lexicon advocates that words are arbitrary labels, and their links to the world are the result of cultural transmission (Bloomfield, 1994; Firth, 1964; Hockett, 1960; Levelt, Roelofs, & Meyer, 1999; Saussure, 1964). In recent years, however, cognitive science is witnessing a substantial paradigm shift in the way the structure of the vocabulary is conceived. The assumption of an arbitrary relationship between phonology and semantics is being progressively reconsidered in favor of a more complex theoretical panorama, where the correspondence between these two domains is carefully examined and not simply rejected a priori (see for instance A. L. Thompson and Do, 2019).

A form of non-arbitrariness in language is iconicity, i.e., a relationship between linguistic sounds and their referents that is defined not only by convention, but also by the sounds’ and the objects’ inherent qualities. For a word to be iconic, these qualities must be related by means of perceptuomotor analogies (Dingemanse, Blasi, Lupyan, Christiansen, & Monaghan, 2015). Historically, early research has focused on vision-related iconicity (namely the association of certain linguistic sounds with some visual attributes of their referents such as their shape or size; see for instance Köhler, 1929, 1947; Maurer, Pathman, & Mondloch, 2006; Sapir, 1929; Werner, 1948). However, more recently, several studies have investigated the relationship between word sounds and perceptual information from other sensory modalities (Fontana, 2013; Fryer, Freeman, & Pring, 2014; Gallace, Boschin, & Spence, 2011; Graven & Desebrock, 2018; Joo, 2020; Speed, Atkinson, Wnuk, & Majid, 2021).

The fact that vision-related semantic information has drawn a lot of attention in iconicity research is to some extent motivated by the largely undisputed predominance of the visual modality in the human perceptual system (Lynott, Connell, Brysbaert, Brand, & Carney, 2020a; Speed & Brybaert, 2022; Vergallito, Petilli, & Marelli, 2020). However, auditory iconicity displays some peculiar properties that make it a worthy testbed for the study of non-arbitrariness. In contrast to the other modalities, auditory iconicity takes place *within* a perceptual modality, associating verbal and non-verbal sounds. Indeed, the most unmistakable cases of iconicity in oral languages are onomatopoetic words, such as *whisper*, *bubble* or *crack*; these expressions are linked to their meaning by a direct imitative relationship and do not involve any cross-modal correspondence. It is thus not surprising to find that auditory iconic words (i.e., lexical items that resemble in their phonological profile the sound associated with their referents, such as *crack*; henceforth AIWs) hold a privileged role within the iconic lexicon. The most iconic words in English and Spanish often have a dominant auditory meaning (e.g., “trumpet”), as documented by explicit iconicity ratings (Perlman, Little, Thompson, & Thompson, 2018; Winter, Perlman, Perry, & Lupyan, 2017). Furthermore, when participants are asked to associate words with their meaning in languages unknown to them, they achieve the highest accuracy with words with high auditory strength (Dingemanse, Schuerman, Reinisch, Tufvesson, & Mitterer, 2016). AIWs have also been shown to be more phonologically marked than other iconic words, exhibiting less sequential predictability than other iconic words in Cantonese (Thompson, Chan, Yeung, & Do, 2022). Additionally, Edmiston, Perlman, and Lupyan (2018) have shown that repeated imitation of environmental sounds can give rise to more word-like forms while retaining a resemblance to the original sounds, highlighting the role of human vocal imitation in the origins of spoken words.

Another piece of evidence for the peculiar status of AIWs comes from linguistic typology, and more precisely from a striking consistency in the cross-lingual distribution of ideophones. Ideophones are a subset of the vocabulary composed of marked words depicting perceptual imagery (Dingemanse, 2012); they constitute sound-symbolic inventories typical of Sub-Saharan African, East Asian or Native American languages. Auditory iconic words constitute the most prominent class of perceptual terms in the ideophonic lexicon across languages. If a language develops ideophones, auditory ideophones will be part of the ideophonic lexicon – in other words, if a language has visual ideophones it therefore must have sound ideophones as well (Dingemanse, 2012). This regularity has led different researchers to set auditory ideophones at the top of the cross-linguistic implicational hierarchy of the ideophonic lexicon (Dingemanse et al., 2015; McLean, 2021).

### Iconicity in cognition

Historically, research on iconicity has often focused on circumscribed phenomena (see Magnus, 2013 for an overview). In recent years, however, the topic has been integrated into broader theories of language evolution (Cabrera, 2012; Dingemanse, Torreira, & Enfield, 2013; Perniss & Vigliocco, 2014; Ramachandran & Hubbard, 2001), acquisition (Asano et al., 2015; Imai, Kita, Nagumo, & Okada, 2008; Murgiano, Motamedi, & Vigliocco, 2020; Perniss & Vigliocco, 2014; see Laing 2014; Laing, 2019 for the role of auditory iconicity in language learning), and processing (Cnudde, Sidhu, & Pexman, 2020; Sidhu, Vigliocco, & Pexman, 2020; Peeters, 2016; Perniss & Vigliocco, 2014; Van Hoey, Thompson, Do, & Dingemanse, 2023). Given the increasing recognition of the role played by iconicity in cognition, it is crucial to devise an appropriate measurement for the construct being studied. The studies hereby discussed operationalized iconicity through subjective ratings; however, iconicity ratings have been criticized for having low construct validity. The ability of language users to judge the fit of a sound with respect to its referent has been questioned, as it has been shown that participants have a positive bias when judging whether sounds fit their referents in their native language (Sutherland & Cimpian, 2015). Furthermore, Thompson, Akita, and Do (2020) have proposed that, for words that are not obviously iconic, participants might base their responses according to sensory information alone as opposed to form-referent resemblance. The inability of participants to judge the iconicity of non-ideophonic words would call for a different, and possibly objective measure of iconicity. Winter and Perlman (2021) provided a response to those criticisms, noting that iconicity ratings have served an important purpose in explaining the distribution of iconic properties in the lexicon; however, they acknowledge that iconicity ratings should be complemented by other tools and methodologies in order to grant a fuller picture on the role of iconicity in language (see also McLean, Dunn, & Dingemanse, 2023).

More generally, the discussion on iconicity ratings can be framed within a broader debate on the role of introspection in psychology and cognitive science. While in the psychological literature it is common practice to employ explicit ratings as independent variables to predict behavioral data (see for instance Sidhu et al., 2020 in the specific case of iconicity ratings), human ratings are themselves an interesting cognitive measure grounded in introspection, that needs to be explained, ideally starting from the objective and measurable properties of the stimuli (see for instance Günther, Petilli, Vergallito, & Marelli, 2020; Günther, Marelli, Tureski, & Petilli, 2021; Westbury, 2016).

### Data-driven measurements in cognitive science

Computationally specified alternatives to human ratings do exist, and they have been proven successful in predicting human performance across a variety of linguistic tasks (see Günther, Rinaldi, and Marelli 2019 for a review). Needless to say, text-based data-driven models and measurements have a long-standing tradition in experimental psychology, computational linguistics and natural language processing (see for instance Landauer & Dumais, 1997; Lund & Burgess, 1996; Mikolov, Chen, Corrado, & Dean, 2013), where they have already been employed to study non-arbitrariness in language (Abramova & Fernández, 2016; Abramova, Fernández, & Sangati, 2013; Dautriche, Mahowald, Gibson, & Piantadosi, 2017; de Varda & Strapparava, 2021; Gutiérrez, Levy, & Bergen, 2016; Shillcock, Kirby, McDonald, & Brew, 2001; Tamariz, 2008). However, language-centered semantic models are not sufficient to capture the phenomenon under scrutiny. The kind of iconic relationship that we study, named in the semiotic literature as “imaginal iconicity” (Nöth, 1999), implies direct similarity between linguistic signs and referred objects. Language-based semantic models, being developed based on linguistic data alone, do not have direct access to the physical properties of the referents. Hence, one of the two semiotic components of the iconic link is not accessible to the model in the first place. For this reason, the objectives of our study required the employment of perceptually grounded models, which have direct access to the physical properties of the referents.

The employment of data-driven models in the study of perceptually-grounded meanings has gained increasing popularity in the last years, with a prominent example being set by the adoption of computer-vision deep-learning architectures in experimental psychology (Günther et al., 2020, 2021; Petilli, Günther, Vergallito, Ciapparelli, & Marelli, 2021) and neuroscience (Seeliger et al., 2018; Yamins & DiCarlo, 2016). These models have also been employed in iconicity research: de Varda and Strapparava (2022) have employed an image-processing neural network to show that word sounds bear a cross-modal resemblance to the visual representations of their referents, with this resemblance being consistent across languages. However, their study was focused on phonovisual iconicity, and thus it implied a cross-modal language-to-perception mapping. This methodology, while overcoming the limitations of iconicity ratings, presents a different shortcoming. Similarly to other data-driven approaches to phonosymbolism (see for instance Blasi, Wichmann, Hammarström, Stadler, & Christiansen, 2016), de Varda and Strapparava ’s (2022) study embraces a *functional* definition of iconicity, characterized as a feature of a signal that allows its meaning to be predicted from its form (Motamedi, Little, Nielsen, & Sulik, 2019). Functional approaches which do not employ direct resemblance as a criterion might be problematic, as they may conflate iconicity and systematicity, a related but distinct phenomenon (Dingemanse et al., 2015). Shifting the focus to auditory iconicity grants the possibility of directly projecting linguistic and perceptual representations onto a shared sound space, without the need of any post hoc transformation. Ultimately, this choice allows us to operationalize iconicity as direct sound resemblance.

**Fig. 1:**
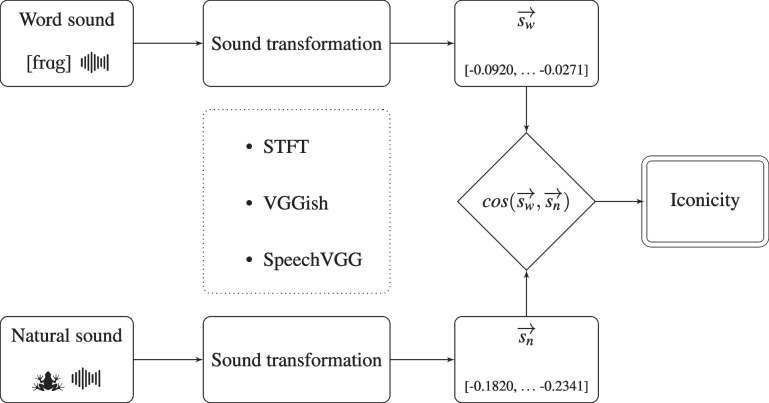
Graphical summary of the experimental pipeline. We embed word sounds (*top*) and natural sounds (*bottom*) into a shared vector format; then, we compute the cosine of the angle subtended by the two vector, and employ this index as a measure of iconicity

In this article, we follow this promising research line by employing different computational models developed in the field of sound engineering as tools for obtaining data-driven, theory-agnostic iconicity measurements. We then use these derived metrics to assess the pervasiveness of iconicity in the English auditory lexicon, aiming to overcome the limitations of iconicity ratings and other data-driven approaches. Then, we empirically validate our measures in explaining human intuitions against a strong baseline of psycholinguistic variables associated with the construct under scrutiny. Finally, we assess the extent to which our results depend on words that are identified as onomatopoetic by English native speakers.

## Methods

In this article, we operationalize auditory iconicity as the objective similarity between (i) the sound of a word and (ii) the natural sounds associated to its referent. We consider a word as auditorily iconic if its phonetic profile – for instance, the phonetic sequence  , “frog” – resembles the natural sounds associated with its referent – for instance, the croaking of a frog. Both word and natural sounds are transposed in a vector format in one of three ways:

1. Short-time Fourier transform (STFT), a procedure that decomposes sound signals into individual frequencies and their amplitudes.
2. Sound classification network, a neural-network model trained to label sounds.
3. Speech recognition network, a neural-network model trained to recognize spoken words.

For all these three methods, the obtained sound representations end up embedding natural sounds and word sounds into a shared vector space. This allows to estimate the similarity between the vector representation of the sound of a word ($\overrightarrow{s_w}$) and its corresponding natural sound ($\overrightarrow{s_n}$), which we employ as a measure of iconicity (Fig. 1). Note that the three representational alternatives are intended as replications, as the same analyses were carried out independently with vector representations obtained with each of them.

The first option that we consider builds on the spectrotemporal features of vocal and natural sounds. A sound signal is composed of several single-frequency sound waves, which can be decomposed through the application of a STFT (Allen, 1977). The Fourier transform converts the signal from the time domain to the frequency domain, comparing the sound signal with sinusoids of various frequencies. The STFT performs this operation on various sequential windows of the audio segment, and the result is called a *spectrum*, which can be visualized through a heatmap as a spectrogram. This is the simplest sound representations we consider in our study, and also constitutes a pre-processing stage of the two neural-network models. In this study, we computed sound spectra utilizing the default parameters that were used in the pre-processing stages of SpeechVGG. In particular, we converted the audio files to mono and resampled them to a sample rate of 16,200 Hz. If the length of the resampled audio was shorter than the target sample rate, the file was padded. Otherwise, the central segment of the audio with a length equal to the target sample rate was extracted. We then computed a STFT on the audio segment using a Hamming window with 256 samples per segment and 128 samples overlap. The resulting matrix was then flattened into a one-dimensional array, which was returned as the final feature vector.

**Fig. 2:**
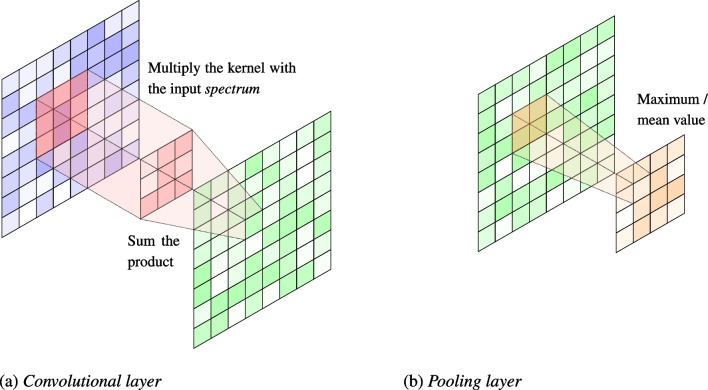
Graphical summary of the operations carried out by the convolutional and the pooling layer of the HCNNs. During convolution, the kernel (*in red*) slides across the input spectrum (*in blue*). At each position, the kernel is multiplied element-wise with the portion of the spectrum it is currently covering, and the resulting product is summed to produce a single output value for that position in the output feature map (*in green*). Pooling (*in orange*) simply involves taking the average or the maximum value out of a set of adjacent units in a feature map

While the human auditory system excels in processing spectrotemporal sound features, sound and voice perception show some degree of invariance to contextually irrelevant changes in the acoustic properties of the stimuli (Ley, Vroomen, & Formisano, 2014). Unless a word is uttered twice in the same way and in an identical environment, the raw sound features corresponding to its pronunciation will be different every time it is produced. Similarly, there is some degree of variation in the raw spectrotemporal features associated with natural sounds. For instance, small and large dogs usually produce high- and low-pitch barks, respectively (Edmiston & Lupyan, 2015). Nonetheless, humans are able to correctly recognize both natural and vocal sounds heard in different auditory contexts, showing the ability to map acoustically different sounds onto similar perceptual representations (Ley et al., 2014). One way to model some degree of invariance with respect to irrelevant low-level features is to employ deep learning architectures and transform the input signal into meaningful representations. Furthermore, audio-processing neural networks have been shown to map onto brain responses to sounds, speaking in favor of their validity as models of human auditory processing (Kell, Yamins, Shook, Norman-Haignere, & McDermott, 2018; see below for a more in-depth discussion). Hence, we complement our spectral baseline with audio representations obtained through two pre-trained hierarchical convolutional neural networks (HCNN) proposed in the field of audio engineering and computer audition.

HCNNs were originally developed for computer vision applications, where they excel at several tasks such as image classification (Krizhevsky, Sutskever, & Hinton, 2012); recently, they have been adapted to process sound data in order to classify environmental sounds (Hershey et al., 2016) and human speech (Beckmann, Kegler, Saltini, & Cernak, 2019). HCNNs exploit the hierarchical nature of the visual and auditory data to assemble representations of increasing abstraction using small and simple kernels (or filters) repeated across the input. These models are generally structured as a sequence of convolutional blocks, followed by standard fully connected layers (see for instance Simonyan & Zisserman, 2015). Convolutional blocks are in turn composed of convolutional and pooling layers. Convolutional layers are the core components of the network; they apply relatively small learned kernels to input images and sound *spectra* by sliding them across the input’s height and width; the dot product between every element of the filter and the input is then calculated at every spatial position, and the output of these operations is called a *feature map* (see Fig. 2a). Pooling layers lower the resolution of the obtained feature maps, taking for instance the average or the maximum activation value from a set of adjacent units, thus creating an invariance to small distortions and shifts (LeCun, Bengio, & Hinton, 2015; see Fig. 2b).

When passing through the sequence of layers, the information in input becomes progressively abstracted away from its superficial features. In auditory HCNNs, the first layers respond to shallow acoustic properties such as loudness and scale, and deeper layers show increased sensitivity to high-level features such as roughness and event categories (Huang, Slaney, & Elhilali, 2018). We employed the output of the convolutional network as an approximation of a representational format proper of the human perceptual system. Sound recognition tasks such as the ones humans perform in everyday life impose a strong functional pressure on the auditory system, and it has been shown that a model trained to perform the same tasks converges to cognitively accurate representational transformations (Kell et al., 2018). Indeed, auditory HCNN-based representations can be successfully mapped onto neural responses to auditory stimuli at different processing stages of the auditory cortex, where intermediate model layers provide the best fit for primary auditory cortex, while deeper layers best predict non-primary responses (Kell et al., 2018). From a behavioral perspective, auditory HCNNs are able to classify sound data with human-level accuracy, and their error patterns resemble those of humans despite not being optimized to do so (Kell et al., 2018). HCNN-based representations have been proposed to be cognitively plausible at least at the computational level of description (Marr, 1982), being able to predict human behavior and performance in several tasks (although these results were primarily based on image-processing HCNNs, see Günther et al. 2020; Günther et al. 2021; Petilli et al. 2021).

**Fig. 3:**
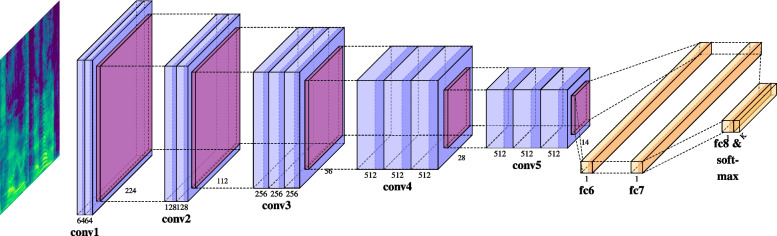
Graphical depiction of the SpeechVGG architecture. The image in input represents a spectrogram; the input is passed through a sequence of convolutional (*light blue*), ReLU (*blue*), pooling (*purple*) and fully connected layers (*yellow*) in order to obtain the output representations. Figure drawn with the PlotNeuralNet package (Iqbal, 2020)

No auditory HCNN, to our knowledge, has been simultaneously trained to recognize spoken words and natural sounds. However, different architectures have been developed separately to carry out one of the two tasks. Interestingly, the VGG16 model, originally developed for large-scale image recognition (Simonyan & Zisserman, 2015), has been adapted by different research groups to recognize words on the basis of their acoustic properties (SpeechVGG; Beckmann et al., 2019), and sounds more generally (VGGish; Hershey et al., 2016). Hence, we chose to employ both architectures in our experimental procedure. SpeechVGG and VGGish are trained to classify sounds, encoded as spectral features, according to their gold-standard labels. In the case of SpeechVGG, these labels are composed by words – for instance, a training instance might be formed by the sound spectrum corresponding to someone pronouncing the word “dog”, and the target category label corresponding to the word. In the case of VGGish, the labels correspond to sound event categories; a training instance might thus be composed of the roar of an engine, that the network should classify as the sound of a car. Crucially, after the training phase, these models are able to correctly classify sounds that were not observed during training, and produce quantitatively defined and semantically meaningful representations for a theoretically infinite range of sounds.

The two models are very similar with respect to their structural configuration, the format of the input they are fed with, and the error function they are trained to minimize. They are composed of a stack of convolutional (with small 3 $\times$ 3 filter size) and max-pooling (with 2 $\times$ 2 window size) layers equipped with ReLU non-linearities; they receive as input log-mel spectrograms and are trained with cross-entropy loss. There are, however, some differences between the two models: while VGGish drops the fifth convolutional block, SpeechVGG maintains the five-block structure of VGG-16. The two fully connected layers at the end of the processing stream have 4,096 and 120 units in VGGish, and a task-dependent shape^1^ in SpeechVGG. Regardless of their architectural differences, which are not of interest for the purpose of this study, both networks produce fixed-length vector representations in response to the sound *spectra* they are fed with. A schematic representation of the SpeechVGG architecture is reported in Fig. 3; for a detailed description, we redirect the reader to the original articles (Beckmann et al., 2019; Hershey et al., 2016; see Simonyan and Zisserman 2015 for the details of the original image-processing VGG16).

### Data

Spoken word data consisted of high-quality recordings released by Tucker et al. (2019) via the Massive Auditory Lexical Decision database (MALD), where they were included as stimuli for a megastudy in auditory lexical decision. Words in the MALD database were selected from different sources, including conversational and written corpora, to ensure generalization across the spoken English lexicon; the recording was performed with professional studio equipment in a sound-isolated room.

For a word to be auditorily iconic, its meaning must be grounded in the auditory modality, as it is necessary for the corresponding concept to be associated with non-linguistic auditory information. A word like “clap” is auditorily iconic since its phonetic realization resembles the sound accompanying a strike of the palms; however, the acoustic information associated with this concept could not be imitated by the phonetics of the corresponding word if that acoustic information was not present in the first place. For this reason, we pre-selected from the dataset the words that exhibited a high auditory perceptual strength (employing the sensory modality ratings released by Lynott, Connell, Brysbaert, Brand, and Carney 2020b). More precisely, we only included items with auditory strength higher than 3.56 in a five-point scale (1.5 SD above the mean; *N* = 374; 10.34% of the words in the MALD database for which the auditory ratings were available). We then scraped Freesound,^2^ a collaborative repository of audio samples where sounds are uploaded to the website by its users, and cover a wide range of subjects, from field recordings and background noises to synthesized sounds. In our scraping procedure, we searched for at least ten sounds tagged with each of the words that we previously selected. In our search, we enforced five constraints: (a) a maximum length of 15 seconds; (b) a maximum of 10 tags per file; (c) a minimum quality rating of 4 out of 5; and (d) a minimum of 500 downloads. These criteria were adopted to automatically select sound events that were sufficiently specific with respect to the tag being considered (a, b) and met a reasonable quality standard, assessed with a sufficient sample size (c, d).

Some of the recordings scraped from Freesound were found to be problematic in relation to our experimental approach.^3^ For instance, some audio files for a target word (e.g., “no”) consisted of a recording of a person uttering that word. As in the present paper, we operationalize auditory iconicity as the resemblance between word and natural sounds, if the natural sound is itself a word sound, the iconicity estimate will be artificially inflated. Furthermore, despite our efforts to ensure a high-quality standard for our audio sample, some natural sounds were found not to contain the sound of the referent (for instance, a sound labeled as “bird” containing a synthetic, high-pitch sound). Thus, two annotators (a research assistant and an intern) were asked to listen to all the sounds in our dataset (*N* = 7401) and remove the items that (a) contained a recording of a person uttering the target word, or (b) did not contain the actual sound of the word’s referent. In total, 1100 out of 7401 sounds were eliminated from the final version of the dataset (14.86%). Out of these 1100 recordings, 68 were eliminated for reason (a), and 1032 were eliminated for reason (b) (0.91% and 13.94% of the total, respectively). Following an additional check of the first author, 12 other sounds were eliminated. Of the 374 word sounds considered at the beginning of the study, 7 did not contain any sound of their referent after the cleaning procedure was performed, and were thus excluded from the following analyses. The final dataset comprised 367 word sounds and 6,289 natural sounds.

The sound vectors associated with these stimuli were derived in the same way for both linguistic (from MALD) and non-linguistic samples (from Freesound). In the pre-processing stages, we averaged the two channels of stereo audio files and resampled them at 16K Hz (the frequency used during pre-training). Then, we computed the sound spectra using the specific parameters that were employed during the pre-training of each network. We fed each sound item *x* in our stimulus set (both word sounds and natural sounds) to the two VGG16-like models, in order to extract the resulting feature map $\varphi (x)$. Note that the pre-trained models were set in evaluation mode, and their parameters were not updated, as no further training was required. While VGGish has a compact 128-dimensional fully connected layer, the fully connected layers of SpeechVGG are task-dependent, and thus are not included in the general-purpose model release, which only returns the output up to the last max-pooling layer. Hence, in the case of VGGish we directly employed $\varphi (x)$ as compact sound representation, whereas in the case of SpeechVGG we extracted the output of block5_pool, flattened it, and reduced it to a 128-dimensional vector applying a PCA transformation.

The outputs of the two models could not be directly compared: the different weights learned during pre-training and the different number of layers cause the sounds in input to be projected onto different sound spaces. Similarly, the sound spectra could not be directly compared with the HCNN-based sound vectors, as the arrays have different shapes and reflect different stages of processing. Hence, we performed all our experiments three times, focusing separately on (i) the spectrotemporal features derived through the STFT, (ii) the distributed representations induced via VGGish, and (iii) the distributed representations induced via SpeechVGG. With considering both a sound- and a speech-processing network we aim at probing the potential advantages of either method: in principle, we would expect the former model (VGGish) to be more sensitive to meaningful properties of natural sounds, and the latter (SpeechVGG) to be optimally receptive to phonetic distinctions. Additionally, the spectral baseline can be informative in terms of the degree of abstraction from low-level features that is necessary to detect iconicity in language.

### Analyses

Once the sound representations were obtained, we computed the similarity between word-sound vectors ($\overrightarrow{s_w}$, e.g., the pronunciation of the word “frog”,  ) and the corresponding natural-sounds vectors ($\overrightarrow{s_n}$, e.g., the croaking of a frog). It is standard practice to employ as a similarity score in high dimensional spaces the cosine similarity between two vectors. The cosine of the angle ${\uptheta }$ subtended by two vectors $\overrightarrow{s_w}$ and $\overrightarrow{s_n}$ is computed as follows:

1 $$\begin{aligned} cos(\uptheta ) = \frac{\overrightarrow{s_w} \cdot \overrightarrow{s_n}}{||\overrightarrow{s_w}|| \cdot ||\overrightarrow{s_n}||} = \frac{\sum _{i=1}^{N}{s_{wi}}{s_{ni}}}{\sqrt{\sum _{i=1}^{N}s_{wi}^{2}}\sqrt{\sum _{i=1}^{N}s_{ni}^{2}}} \end{aligned}$$

Where $\overrightarrow{s_{wi}}$ and $\overrightarrow{s_{ni}}$ are components of the vectors $\overrightarrow{s_w}$ and $\overrightarrow{s_n}$, respectively, and *N* corresponds to the dimensionality of the vectors (i.e., 128 in our case). The obtained estimates were tested in two analyses.

First, we employed this similarity metric to assess whether auditory words were more similar to the sounds associated with their referent than to the other sounds in the dataset. For each $\overrightarrow{s_w}$, we computed the cosine similarity with all the $\overrightarrow{s_n}$, including both sounds that were associated to the given word and sounds that were not. We then evaluated whether $\overrightarrow{s_w}$ were more similar to the associated $\overrightarrow{s_n}$ than to other unrelated $\overrightarrow{s_n}$ by dummy-coding whether they matched (0-1), and employing this value as a regressor, with the cosine similarity as the dependent variable. Due to the hierarchical structure of our data (i.e., multiple natural sounds nested within word sounds), we employed linear mixed-effects models, with random slopes and intercepts for both $\overrightarrow{s_w}$ and $\overrightarrow{s_n}$ (in line with the suggestion of fitting maximal models when possible, see Barr, Levy, Scheepers, and Tily 2013). We then dropped the random components of the regression when their variance was equal to zero, to avoid singular fit (see for instance Pasch, Bolker, & Phelps, 2013).

Second, we evaluated the degree of association between our data-driven measures of auditory iconicity and explicit ratings of iconicity obtained by English native speakers, as released via two large norming studies (Winter et al., 2017, 2023). We also tested whether our measures were significant predictors of iconicity ratings against a strong baseline of psycholinguistic variables that have been shown to be associated with such a construct. Indeed, it has been shown that iconicity ratings are negatively associated with word length (Perry, Perlman, & Lupyan, 2015; Winter et al., 2017; Winter et al., 2023), log frequency (Perry et al., 2015; Perry, Perlman, Winter, Massaro, & Lupyan, 2018; Winter et al., 2017, 2023), age of acquisition (Perry et al., 2018; Winter et al., 2023), concreteness (Hinojosa, Haro, Magallares, Duñabeitia, & Ferré, 2021; Winter et al., 2023), and contextual diversity (Lupyan & Winter, 2018); furthermore, they are positively correlated with perceptual experience (Sidhu & Pexman, 2018; Winter et al., 2017, 2023) and phonological markedness (Dingemanse & Thompson, 2020). In our analyses, we included these measures as covariates to test the predictive power of our metrics while controlling for other possible confounds. Data were obtained from available norms of perceptual strength (Lynott et al., 2020b), SUBTLEX-US log frequency and contextual diversity (Brysbaert & New, 2009), concreteness (Brysbaert, Warriner, & Kuperman, 2014), and age of acquisition (Kuperman, Stadthagen-Gonzalez, & Brysbaert, 2012). Phonological markedness was measured with the same word-sound vectors employed to derive our data-driven measurement of iconicity; it was operationalized as the cosine distance of each $\overrightarrow{s_w}$ from the centroid of the word sound space. This choice ensured that the predictive power associated with our iconicity metric was not confounded with phonological markedness.^4^

### Follow-up analysis: removal of onomatopoetic words

As a follow-up analysis, we removed from our dataset all the words that were identified as onomatopoetic forms by two English native speakers, and repeated our analyses on this subset. This follow-up study was aimed at testing whether our results held after excluding the words that were explicitly perceived as imitative.^5^ If imitative patterns could be detected with our computational approach even after the exclusion of words judged as onomatopoetic, this would suggest that subtle iconic patterns in the lexicon can elude human explicit intuitions while still contributing to the phonological structure of the auditory vocabulary. Two native English speakers (graduate students with training in psychology, one American, one British) were presented with the original list of 374 words and asked to indicate whether each word was an instance of onomatopoeia or not. They were instructed to indicate as onomatopoetic words those stimuli that satisfied either of these two conditions:

1. The word phonetically imitates or resembles the sound it describes. According to this first definition, onomatopoeic words sound like the noises or actions they represent, like “buzz” for the sound a bee makes or “splash” for the sound of something falling in the water.
2. The sound of the word resembles a sound associated with the object or action that the word denotes. One example of this is the word “cuckoo”. *Cuckoo* is the bird’s name, but its acoustic resemblance is to the song that it produces, not the bird itself.

The two annotators were further asked to indicate the cases where they were unsure about the answer. To be maximally conservative in our follow-up study, we repeated our analyses after removing all the words that at least one participant indicated as onomatopoetic or was unsure.

Our experimental pipeline is summarized in Fig. 1. All code is publicly available.^6^ The code for extracting the embeddings was adapted from the one available on TensorFlow Hub for VGGish^7^ and from GitHub for SpeechVGG.^8^ Due to copyright constraints, we release aggregated sound vectors for the natural sounds, since the various recordings are protected by different restrictions.

## Results

### Iconicity in the auditory lexicon

We were not able to find an effect of conditions (i.e., a higher similarity between matching versus non-matching $\overrightarrow{s_w}$ and $\overrightarrow{s_n}$) employing the raw spectral representations: the average cosine similarity of matching and non-matching $\overrightarrow{s_w}$ and $\overrightarrow{s_n}$ were virtually indistinguishable (-0.0497, SD = 0.4532 and -0.0440, SD = 0.4438, respectively; B = 0.0068, SE = 0.0048, *t* = 1.4110, *p* = 0.158, *N* = 2,308,063). However, we detected a significant effect of condition employing the more sophisticated sound processing networks. In the case of VGGish, $\overrightarrow{s_w}$ and $\overrightarrow{s_n}$ had a similarity of 0.4180, SD = 0.1498 when they matched, and 0.4111, SD = 0.1516 when they corresponded to different items. The representations obtained from the other network reflected the same distinction: the average similarity between word and natural sounds was -0.0452, SD = 0.1606 when the word was the label of the given sound, and -0.0619, SD = 0.1527 otherwise. The statistical significance of this distinction was confirmed by the results of our mixed-effects models. Indeed, we found a significant effect of condition both in the case of VGGish (B = 0.0033, SE = 0.0015, *t* = 2.176, *p* = 0.0296, *N* = 2,308,063) and SpeechVGG (B = 0.01350, SE = 0.0017, *t* = 7.789, *p* $\ll$ 0.0001, *N* = 2,308,063). The results were obtained by dropping the random slopes from the models – which had variance equal to zero –, but aside from convergence issues the random structure of the model did not affect the parameter estimation, as the fixed effects of condition remained significant. It must, however, be acknowledged that, although significant, the observed effects are small, with numerically reduced differences in means and relatively high variability in the data, and obtained by considering a rather large dataset.

Note that the absolute differences between the mean similarities obtained with the spectra, VGGish, and SpeechVGG are not of interest for the purposes of the study. They simply reflect that the auditory space where $\overrightarrow{s_w}$ and $\overrightarrow{s_n}$ are projected is characterized by different levels of dispersion. Indeed, the average closeness to the centroid,^9^ defined as $\frac{1}{N}\sum _{i=1}^{N}cos(x_i, \bar{x})$, is 0.4835 employing the sound spectra, 0.6644 in the case of VGGish, and 0.0013 with SpeechVGG. This indicates that the sound spectra and VGGish project the sound vectors relatively close to the centroid, resulting in medium-to-high similarity scores between its generated embeddings. Conversely, the opposite holds for SpeechVGG, where the network generates sound vectors that are, on average, nearly orthogonal. Note that the embedding spaces also contained many $\overrightarrow{s_w}$ and $\overrightarrow{s_n}$ pairs that had negative cosine similarity values (VGGish: $< 0.01$%; SpeechVGG: 70.71%; spectra: 60.44%). In the context of standard, text-based distributional semantic models, it has been argued that negative cosine similarity values carry little useful semantic information (Rotaru, Vigliocco, & Frank, 2018) and it is not clear how to interpret them (Günther & Marelli, 2016). While it is unclear whether these considerations also apply to auditory vectors, especially since negative cosine similarity values are so common, we repeated the analyses setting the negative cosine similarity values to zero. This methodological choice had no major impact on our results (see Appendix A).

As a robustness check, we verified that the reported effects in the mixed effects models analyses remained significant with non-parametric testing. To do so, we randomly reassigned the condition variable (i.e., the independent variable of interest indicating whether $\overrightarrow{s_w}$ and $\overrightarrow{s_n}$ corresponded to the same label). For each word sound and natural sound, we randomly selected a pair to be coded as matching, and refitted the mixed effects regression model with the same specification on the new dataset. This procedure was repeated 500 times for each regression model, and empirical *p* values were calculated as the proportion of the models with random condition assignment that obtained an absolute *t* value at least as extreme as the one observed in the corresponding model with proper condition assignment. Empirical *p* values were obtained after adding 1 to both the numerator and the denominator (Davison & Hinkley, 1997). The non-parametric analyses yielded similar results to the parametric ones (spectrum: *p* = 0.1537; VGGish: *p* = 0.0100; SpeechVGG: *p* = 0.0020).

**Table 1:** Five most and least auditorily iconic words according to the metrics based on the sound spectra, VGGish, and SpeechVGG (*Estimate*)

	Spectrum			VGGish			SpeechVGG		
	Word	Estimate	Rating	Word	Estimate	Rating	Word	Estimate	Rating
Most iconic	thud	0.9359	6.3000	announcer	0.7722	3.7272	drip	0.5374	5.6000
	frog	0.9218	3.8000	media	0.7683	2.8000	pop	0.4894	6.4000
	chat	0.9061	4.4000	mumble	0.7584	5.0909	thud	0.4875	6.3000
	click	0.9034	6.7000	vocal	0.7578	3.2727	clunk	0.4788	6.8000
	drip	0.8916	5.6000	speech	0.7563	NA	click	0.4477	6.7000
	$\cdots$	$\cdots$	$\cdots$	$\cdots$	$\cdots$	$\cdots$	$\cdots$	$\cdots$	$\cdots$
Least iconic	beg	–0.6272	4.8000	response	0.1703	NA	trumpet	–0.3778	4.0000
	people	–0.6361	3.4000	story	0.1612	NA	engine	–0.3786	NA
	tune	–0.6495	4.4000	beg	0.1463	4.8000	violin	–0.3864	3.7000
	jet	–0.6644	3.9000	newsflash	0.1211	NA	broadcast	–0.3989	NA
	bellow	–0.6722	4.2000	soundtrack	0.0916	NA	report	–0.3999	2.5000

The column *Rating* reports the average iconicity rating associated to the word (Winter et al., 2023). The complete results are available in the online supplementary materials

**Table 2:** Results of the linear models with the human ratings as dependent variables, the data-driven iconicity estimates as predictors, and the baseline of psycholinguistic variables listed in the Methods section as covariates

		Spectrum					VGGish					SpeechVGG				
	Measure	B	SE	*t*	*p*	$\Delta$R^2^	B	SE	*t*	*p*	$\Delta$R^2^	B	SE	*t*	*p*	$\Delta$R^2^
Winter et al. (2017)	Markedness	–0.564	1.592	–0.354	0.724	0.003	–3.005	3.878	–0.775	0.440	0.003	–0.673	1.718	–0.392	0.696	0.000
	CD	–0.580	0.292	–1.984	0.049	0.115	–0.625	0.294	–2.124	0.035	0.121	–0.560	0.288	–1.946	0.054	0.111
	Auditory	–0.053	0.247	–0.216	0.829	0.000	–0.019	0.254	–0.075	0.940	0.001	0.008	0.245	0.034	0.973	0.001
	Conc.	–0.553	0.147	–3.769	$<.001$	0.059	–0.557	0.150	–3.706	$<.001$	0.058	–0.502	0.147	–3.419	0.001	0.053
	Freq.	0.010	0.560	0.018	0.986	0.107	0.056	0.566	0.099	0.921	0.112	0.020	0.551	0.035	0.972	0.104
	AoA	–0.235	0.072	–3.250	0.001	0.036	–0.230	0.075	–3.075	0.003	0.035	–0.218	0.072	–3.039	0.003	0.033
	Length	–0.074	0.057	–1.295	0.197	0.015	–0.051	0.057	–0.897	0.371	0.012	–0.047	0.056	–0.849	0.397	0.012
	$\underline{\textrm{Iconicity}}$	0.630	0.208	3.027	0.003	0.066	1.667	0.794	2.100	0.037	0.041	2.164	0.594	3.641	$<.001$	0.102
Winter et al. (2023)	Markedness	0.877	0.895	0.980	0.328	0.003	–0.655	2.271	–0.288	0.773	0.000	0.609	1.166	0.522	0.602	0.001
	CD	–0.079	0.233	–0.338	0.736	0.037	–0.124	0.232	–0.533	0.594	0.040	–0.118	0.232	–0.509	0.611	0.038
	Auditory	0.151	0.175	0.865	0.388	0.005	0.141	0.176	0.806	0.421	0.005	0.131	0.175	0.749	0.454	0.005
	Conc.	–0.116	0.089	–1.303	0.194	0.004	–0.095	0.092	–1.032	0.303	0.004	–0.066	0.093	–0.708	0.479	0.004
	Freq.	–0.497	0.457	–1.088	0.278	0.040	–0.453	0.457	–0.991	0.323	0.043	–0.438	0.456	–0.960	0.338	0.041
	AoA	–0.178	0.042	–4.264	$<.001$	0.065	–0.180	0.042	–4.295	$<.001$	0.065	–0.178	0.042	–4.274	$<.001$	0.066
	Length	–0.167	0.036	–4.693	$<.001$	0.087	–0.143	0.034	–4.177	$<.001$	0.079	–0.151	0.035	–4.368	$<.001$	0.081
	Iconicity	0.346	0.148	2.340	0.020	0.028	1.030	0.471	2.185	0.030	0.028	0.948	0.393	2.413	0.017	0.033

The $\Delta$R^2^ is the R^2^ ascribed to the measure of interest, partitioned by averaging over the orders of the predictors (Grömping, 2007)

### Prediction of human ratings

Table 1 reports the five most and least iconic words according to the three representational formats we considered in our study. From a qualitative inspection it appears that both the spectrum- and the SpeechVGG-based iconicity estimates correctly identified onomatopoetic words as the most iconic (e.g., “thud”, “click”); on the other hand, the VGGish-based metric seems to be biased in assigning words referring to vocal sounds high iconicity scores (e.g., “announcer”, “speech”). We speculate that VGGish might assign high similarity scores to vocal sounds due to the task it was trained on (aspecific sound classification), which induced a pressure to represent vocal sounds as similar to each other, regardless of their specific content. If vocal sounds are classified as a single or a few classes in the pre-training tag set, they will be represented similarly. This characteristic of the VGGish model might explain the observed bias in the iconicity scores assigned to words referring to vocal sounds. From a quantitative analysis, it emerged that all three of our iconicity measures displayed a significant correlation with the explicit ratings obtained from human participants in norming studies. More precisely, the spectrum-based similarity scores were significantly correlated with both the ratings provided by Winter et al. (2017) (*r* = 0.2634, *p* = 0.0004, *N* = 175) and the ones released by Winter et al. (2023) (*r* = 0.1755, *p* = 0.0056, *N* = 248). Likewise, the SpeechVGG-based iconicity scores were significantly associated with the ratings obtained in the two norming studies (*r* = 0.3348, *p* $\ll$ 0.0001, *N* = 175; *r* = 0.1672, *p* = 0.0083, *N* = 248), and the same held for the data-driven scores obtained with VGGish (*r* = 0.1885, *p* = 0.0125, *N* = 175, and *r* = 0.1742, *p* = 0.0060, *N* = 248, respectively). Furthermore, the spectrum-based metric was significantly correlated with the similarity scores based on VGGish (*r* = 0.2499, *p* $\ll$ 0.0001, *N* = 367) and SpeechVGG (*r* = 0.4958, *p* $\ll$ 0.0001, *N* = 367), and the two measures we obtained from the two neural architectures were significantly associated (*r* = 0.3969, *p* $\ll$ 0.0001, *N* = 367), indicating that while each method might have been sensitive to different sound properties, they all revealed a coherent pattern of similarity. When the predictive power of our iconicity measurements was assessed against the baseline of psycholinguistic variables described in the previous section, the best predictor of human ratings was the SpeechVGG-based metric, which clearly outperformed the other predictors in both datasets in terms of $\Delta$R^2^ (see Table 2). Furthermore, all three iconicity estimates were significantly associated with human responses across both rating datasets.

### Removal of onomatopoetic words

The two annotators displayed a substantial agreement (as per Cohen’s guidelines; see Cohen 1960) in identifying the onomatopoetic words in our original dataset ($\kappa$ = 0.642, *z* = 12.4, *p* < 0.001, *N* = 374). The words that were identified as onomatopoetic or dubious by at least one annotator were 139 (37.17%).

After the removal of the words judged as onomatopoetic, we were not able to detect a significant effect of condition employing the raw sound spectra (B = 0.0005, SE = 0.0059, *t* = 0.086, *p* = 0.932, *N* = 913,481) nor VGGish (B = 0.0023, SE = 0.0019, *t* = 1.181, *p* = 0.238, *N* = 913,481); however, we did find an effect of condition when employing SpeechVGG-based representations (B = 0.0060, SE = 0.0021, *t* = 2.822, *p* = 0.0048, *N* = 913,481). Non-parametric significance testing led to the same conclusions (spectrum: *p* = 0.9182; VGGish: *p* = 0.2036; SpeechVGG: *p* = 0.0040).

Regarding the prediction of the human ratings, no data-driven iconicity estimate was significantly correlated with the human rating data after onomatopoetic forms were excluded, neither for the ratings provided by Winter et al. (2017) (spectra: *r* = 0.1632, *p* = 0.1181, *N* = 93; VGGish: *r* = -0.0313, *p* = 0.7660, *N* = 93; SpeechVGG: *r* = 0.1129, *p* = 0.2813, *N* = 93) nor for the ones released by Winter et al. (2023) (spectra: *r* = 0.0053, *p* = 0.9456, *N* = 164; VGGish: *r* = -0.0112, *p* = 0.8868, *N* = 164; SpeechVGG: *r* = -0.0976, *p* = 0.214, *N* = 164). The effect did not emerge after controlling for the iconicity-related covariates described in the Methods section, neither in the dataset released by Winter et al. (2017) (spectra: B = 0.197, SE = 0.231, *t* = 0.850, *p* = 0.398, $\Delta$R^2^ =0.020, *N* = 93; VGGish: B = 0.165, SE = 0.773, *t* = 0.214, *p* = 0.831, $\Delta$R^2^ = 0.002, *N* = 93; SpeechVGG: B = 0.305, SE = 0.781, *t* = 0.390, *p* = 0.697, $\Delta$R^2^ = 0.010, *N* = 93) nor Winter et al. (2023) (spectra: B = -0.020, SE = 0.163, *t* = -0.121, *p* = 0.904, $\Delta$R^2^ = 0.000, *N* = 164; VGGish: B = 0.022, SE = 0.487, *t* = 0.046, *p* = 0.964, $\Delta$R^2^ = 0.000, *N* = 164; SpeechVGG: B = -0.402, SE = 0.445, *t* = -0.903, *p* = 0.368, $\Delta$R^2^ = 0.006, *N* = 164).

## Discussion

The present work provides empirical support for the idea of a widespread phonosymbolic substrate underlying the auditory vocabulary: when transformed into an HCNN-based compact vector format, words with high perceptual strength in the auditory modality resemble the sounds of their referent more than what would be expected by chance. Notably, these auditory phonosymbolic patterns can be detected even in English, a language that is known to be iconically impoverished (Nuckolls, 2003). Auditory mimicry thus plays a significant role in the phonetic structure of the auditory lexicon, extending beyond the anecdotal cases offered by self-evident onomatopoetic forms (see also Thompson, Van Hoey, and Do 2021 for similar results in the case of motion ideophones). Indeed, our best-performing model is able to detect signs of imitation in the lexicon even if the words that are perceived as onomatopoetic by human annotators are removed from the analyses. An interesting open question is why the pervasiveness of onomatopoeia has often eluded the intuitions of the philosophers and linguists who studied the nature of linguistic signs. Historically, the role of iconicity in language has been consistently dismissed (Hockett, 1960; Saussure, 1964) or restricted to a merely “not wholly insignificant” (Whitney, 1874, p. 102) or “vanishingly small” (Newmeyer, 1992, p. 758) portion of the lexicon. Even in contemporary iconicity research, most studies have trended towards exploring subjective and indirect characterizations of iconicity. For instance, Winter et al. (2023) defined iconicity as “perceived resemblance” between form and meaning; on a similar vein, Sidhu and Pexman (2018) meticulously differentiated sound symbolism from onomatopoeia as different categories possibly supported by different mechanisms, and focused on the latter in their review. These examples illustrate that recent studies in the iconicity literature emphasize instances of indirect iconicity, without recognizing a core role to direct phonetic mimicry. We speculate that the little attention that has been directed towards imitative iconicity, both historically and in recent years, can be ascribed to the natural limitations to the degree of resemblance that vocal sounds can exhibit with respect to natural sounds.

Indeed, the process of compressing noises into sequences of vowels and consonants must abide by both biological and linguistic constraints. First, the vocal sounds must be pronounceable, consistently with the anatomical structure of the human vocal system (Assaneo, Nichols, & Trevisan, 2011). Second, they must be part of the phonological inventory of a given language (Bredin, 1996). These limitations will inevitably result in an information loss, which might hinder the correspondence between linguistic and non-linguistic sounds, increasing their absolute spectral difference and ultimately making it more difficult to appreciate such correspondences. The employment of objective sound similarity measures can reveal subtle correspondences between natural and vocal sounds that might not be accessible to human intuitions, but are anyway encoded in the phonological profiles of AIWs.

While apparently the sound representations based on the raw spectra were not sufficient to detect the widespread presence of auditory iconicity, both VGGish and SpeechVGG produced sound embeddings that reflected the general correspondence between natural and speech sounds. This is particularly informative since the two networks treat the input sounds in a fundamentally different way: VGGish is accustomed to process words as if they were natural sounds, in line with the type of data it received as input during pre-training; conversely, SpeechVGG handles natural sounds as vocal sounds.^10^ The fact that both networks largely detected the general similarity between congruent natural and vocal sound data strengthens the reliability of our findings, providing converging evidence from two independent representational formats.

Besides attesting the pervasiveness of iconicity in the auditory lexicon, our computational measures were significantly predictive of human judgements on form-meaning resemblance. This result shows that participants that produce iconicity ratings sensitize to the similarity between word sounds and their referents, approximating their distance in an auditory space. On the other hand, the predictivity of our measurements with respect to iconicity ratings only holds if onomatopoetic words are included in the sample, suggesting that, for words that are not obviously onomatopoetic, participants do not base their judgements on sound resemblance. These results are compatible with the proposal by Thompson et al. (2020), who suggested that, for words that are not obviously iconic, participants may not base their responses on form-meaning resemblance (i.e., what iconicity ratings are supposed to measure), but rather on perceptual information, without any link to phonology. However, an alternative interpretation of our findings is that, for words that are not identified as onomatopoetic, participants might base their iconicity judgements on non-auditory properties. Our study focused on a single dimension of similarity, namely auditory resemblance, whereas iconicity ratings are expected to be produced by taking into account perceptual information from multiple modalities. We acknowledge that, in order to fully capture human intuitions about iconicity, future studies will have to consider other sensory dimensions as well.

In our analyses, SpeechVGG consistently outperformed VGGish and the spectral baseline both in detecting iconic patterns in the lexicon and predicting human iconicity ratings across Winter et al. ’s (2017) and Winter et al. ’s (2023) norms. This result suggests that optimizing a network to be maximally sensitive to phonetic distinctions increases its ability to detect iconic patterns in language. We speculate that this difference might be due to the inherent asymmetry in the variability of speech signals and natural sounds. The natural sounds considered in this study covered a wide range of domains, from environmental noises to animal cries and sounds produced by human-created objects; conversely, speech sounds were limited in their variability by the English phonological inventory. A small fluctuation in a speech sound might entail a substantial meaning shift (e.g., “pun”  and “bun”  , which only differ by one subsegmental feature [+/- voiced] in American English); however, it is difficult to conceive two natural sounds that are nearly identical and yet correspond to two radically different objects, which humans are able to distinguish on the basis of their sound. If a model is not optimized to be receptive to speech sounds, small and yet meaningful phonetic differences might be lost in the representations it produces, ultimately affecting the network’s accessibility to auditory iconic patterns.

In the present study, we operationalized the construct of auditory iconicity in its most elemental terms as the objective similarity between the sound of spoken words and natural sounds associated to their referents. This choice entails that our study has high construct validity, and indicates that the correspondences we detected are inherently iconic. Conversely, construct validity is often weaker when adopting other experimental designs. Indeed, a common difficulty in iconicity research is to disentangle iconic biases, i.e., analogical and perception-related correspondences, from other forms of non-arbitrariness. For instance, phonosemantic regularities can be an instantiation of systematicity, i.e., statistical regularities in form-meaning relationships that are not meaningful in themselves, but are a property of a linguistic system as a whole (Dingemanse et al., 2015; Murgiano, Motamedi, & Vigliocco, 2021; Thompson & Do, 2019). Another alternative to iconicity is indexicality, a form of non-arbitrariness that relates perceptual experience (e.g., a slap) and words (e.g., *ouch*) on account of their co-occurrence, without any imitative link (Dingemanse, 2021). While discerning between these alternatives is often problematic, our experimental setting measures within-modality direct resemblance, mitigating this theoretical complication. Note, however, that we cannot exclude the possibility that our findings might have been partially driven by indirect iconicity.^11^ Consider for instance the case of bird names. Larger birds tend to sing deeper songs (Ryan & Brenowitz, 1985); in this case, if bird names were dependent on their size, the resemblance between bird names and their associated sounds would be mediated by size sound symbolism, and not sound imitation. We believe that the qualitative examples we reported suggest that, at least for SpeechVGG and the sound spectra, the iconic associations we detected with our approach reflect direct sound imitation, as the words that are most iconic according to our estimates are onomatopoetic. However, we leave to future research a thorough analysis of the impact of non-imitative indirect iconicity on data-driven estimates of sound resemblance.

## Conclusion

Several studies have inspected the relationship between iconicity ratings and different semantic dimensions, such as concreteness (Hinojosa et al., 2021; Winter et al., 2023) and perceptual experience (Sidhu & Pexman, 2018; Winter et al., 2017, 2023). Perhaps surprisingly, no study to our knowledge has ever assessed the relationship between iconicity ratings and the construct they are expected to measure, namely perceptual resemblance. This is precisely what we empirically tested in this article. With this respect, our study yielded mixed results: iconicity ratings appear to be reliable in identifying onomatopoetic forms, which drive the correlation between data-driven and human-annotated iconicity estimates. When the similarity between word and natural sounds is not evident, iconicity ratings are not approximated by objective measurements of sound resemblance. Future research is needed to understand the factors that explain iconicity ratings in those conditions, taking into account non-auditory semantic features. At the same time, our study demonstrates that data-driven alternatives to human judgments do exist, and can be employed to study subtle and elusive phenomena such as iconicity.

## Data Availability

Due to copyright constraints, we release aggregated sound vectors for the natural sounds, since the various recordings are protected by different restrictions.

## Appendix A: Negative cosine similarities

As mentioned in the Results section, it has been argued that negative cosine similarity values between distributional semantic vectors are difficult to interpret (Günther & Marelli, 2016) and provide little semantic information (Rotaru et al., 2018). Thus, we wanted to ensure that the conclusions that we drew from our analyses did not critically depend on negative cosine similarity values. We replicated both the analysis on the pervasiveness of iconicity in the auditory lexicon and the prediction of iconicity ratings after setting the negative cosine similarity values to zero. The choice of whether to consider the negative cosines had little impact on our results.

Concerning our analysis on the pervasiveness of iconicity in the auditory lexicon, we were not able to find an effect of condition when employing the raw sound spectra (B = 0.0026, SE = 0.003, *t* = 0.877, *p* = 0.381, *N* = 2,308,063), but only when considering VGGish- (B = 0.0033, SE = 0.0015, *t* = 2.176, *p* = 0.0296, *N* = 2,308,063) and SpeechVGG-based (B = 0.0069, SE = 0.001, *t* = 6.834, *p* $\ll$ 0.0001, *N* = 2,308,063) sound representations.

Even with negative cosine values set to zero, our estimates were significantly correlated with the human ratings in the two datasets considered in this study. The spectrum-based similarity scores were significantly correlated with both the ratings provided by Winter et al. (2017) (*r* = 0.2773, *p* = 0.0002, *N* = 175) and the ones released by Winter et al. (2023) (*r* = 0.2069, *p* = 0.001, *N* = 248). Similarly, the SpeechVGG-based iconicity estimates were correlated with the ratings obtained in the two rating datasets (*r* = 0.3256, *p* $\ll$ 0.0001, *N* = 175; *r* = 0.178, *p* = 0.0049, *N* = 248), and the same held for the VGGish-based scores (*r* = 0.1885, *p* = 0.0125, *N* = 175, and *r* = 0.1742, *p* = 0.0060, *N* = 248). Furthermore, the spectrum-based iconicity measure was correlated with both the similarity scores based on VGGish (*r* = 0.2078, *p* $\ll$ 0.0001, *N* = 367) and based on SpeechVGG (*r* = 0.4564, *p* $\ll$ 0.0001, *N* = 367), and the two measures we obtained from the two neural networks were significantly associated (*r* = 0.2466, *p* $\ll$ 0.0001, *N* = 367). When controlling for the baseline of psycholinguistic covariates described in the Methods section, all three iconicity estimates were significantly associated with human responses across both rating datasets (see Table 3), and, as in our main analyses, the best predictor of human ratings (in terms of $\Delta$R^2^) was the SpeechVGG-based metric.

**Table 3:** Results of the linear models predicting the human ratings from the data-driven iconicity estimates as predictors, after setting to zero the negative cosine similarity values

		Spectrum					VGGish					SpeechVGG				
	Measure	B	SE	*t*	*p*	$\Delta$R^2^	B	SE	*t*	*p*	$\Delta$R^2^	B	SE	*t*	*p*	$\Delta$R^2^
Winter et al. (2017)	Markedness	–0.449	1.582	–0.284	0.777	0.002	–3.005	3.878	–0.775	0.440	0.003	0.016	1.702	0.009	0.993	0.000
	CD	–0.610	0.291	–2.098	0.038	0.116	–0.625	0.294	–2.124	0.035	0.121	–0.571	0.285	–2.000	0.047	0.112
	Auditory	–0.04	0.247	–0.161	0.872	0.000	–0.019	0.254	–0.075	0.940	0.001	–0.075	0.242	–0.312	0.756	0.000
	Conc.	–0.544	0.146	–3.719	$<.001$	0.058	–0.557	0.15	–3.706	$<.001$	0.058	–0.564	0.141	–3.991	$<.001$	0.061
	Freq.	0.063	0.558	0.113	0.911	0.108	0.056	0.566	0.099	0.921	0.112	0.015	0.548	0.028	0.978	0.105
	AoA	–0.235	0.072	–3.276	0.001	0.036	–0.230	0.075	–3.075	0.003	0.035	–0.242	0.070	–3.435	0.001	0.037
	Length	–0.065	0.057	–1.134	0.259	0.014	–0.051	0.057	–0.897	0.371	0.012	–0.042	0.056	–0.763	0.447	0.012
	$\underline{\textrm{Iconicity}}$	1.032	0.323	3.193	0.002	0.071	1.667	0.794	2.100	0.037	0.041	3.965	1.007	3.939	$<.001$	0.097
Winter et al. (2021)	Markedness	1.027	0.896	1.147	0.253	0.003	–0.655	2.271	–0.288	0.773	0.000	0.858	1.161	0.739	0.461	0.002
	CD	–0.089	0.233	–0.380	0.704	0.037	–0.124	0.232	–0.533	0.594	0.040	–0.131	0.231	–0.567	0.572	0.038
	Auditory	0.146	0.175	0.837	0.404	0.005	0.141	0.176	0.806	0.421	0.005	0.102	0.174	0.589	0.557	0.004
	Conc.	–0.117	0.089	–1.313	0.190	0.004	–0.095	0.092	–1.032	0.303	0.004	–0.081	0.090	–0.899	0.369	0.004
	Freq.	–0.478	0.457	–1.046	0.297	0.040	–0.453	0.457	–0.991	0.323	0.043	–0.418	0.454	–0.920	0.359	0.041
	AoA	–0.176	0.042	–4.222	$<.001$	0.064	–0.18	0.042	–4.295	$<.001$	0.065	–0.18	0.041	–4.361	$<.001$	0.067
	Length	–0.163	0.036	–4.594	$<.001$	0.085	–0.143	0.034	–4.177	$<.001$	0.079	–0.157	0.034	–4.573	$<.001$	0.085
	$\underline{\textrm{Iconicity}}$	0.542	0.231	2.350	0.020	0.032	1.030	0.471	2.185	0.030	0.028	1.926	0.687	2.802	0.006	0.035

## Footnotes

1 The final fully-connected and output layers of the SpeechVGG model can be altered based on the specific application for which the model is being used, such as language identification or speech inpainting.

2 Accessed at https://freesound.org/; for the scraping, we adapted the scripts available at https://github.com/cchyung/freesound-scraper

3 We thank an anonymous reviewer for bringing this issue to our attention.

4 We thank an anonymous reviewer for bringing to our attention this possible confound.

5 We thank Gary Lupyan for suggesting this analysis.

6 https://github.com/Andrea-de-Varda/iconicity-datadriven

7 https://tfhub.dev/google/vggish/1

8 https://github.com/bepierre/SpeechVGG/blob/master/examples/speech_music_noise/extract_and_classify.ipynb

9 We could not employ more common dispersion measures such as the standard deviation since distances in high-dimensional spaces are usually measured with the cosine similarity. This measure is defined in the range (–1, 1), and there is a meaningful difference between a positive and a negative cosine similarity between two vectors, which would be lost by squaring.

10 Note that while processing natural sounds with a speech network (and vice versa) might increase the average similarity between speech and natural sounds with respect to employing the raw sound spectra, our results do not depend on these similarities alone, but on the similarities of matching versus non-matching natural and word sounds. We thank an anonymous reviewer for giving us the opportunity to clarify this point.

11 We thank an anonymous reviewer for raising this problem and suggesting the relevant example that follows.

## Code availability

All code is publicly available(https://github.com/Andrea-de-Varda/iconicity-datadriven). The code for extracting the embeddings was adapted from the one available on TensorFlow Hub for VGGish(https://tfhub.dev/google/vggish/1) and from GitHub for SpeechVGG(https://github.com/bepierre/SpeechVGG/blob/master/examples/speech_music_noise/extract_and_classify.ipynb).
